# Short-Chain Fatty Acids: Bridging Gut Microbiota and Systemic Aging—Mechanisms, Interventions, and Current Challenges

**DOI:** 10.3390/metabo16070438

**Published:** 2026-06-23

**Authors:** Pengpeng Xie, Yaoye Pei, Luyun Xu, Yuanhao Shan, Xiamin Cao

**Affiliations:** 1 School of Life Sciences, Soochow University, Suzhou 215123, China; 2330401088@stu.suda.edu.cn (P.X.); 2330401049@stu.suda.edu.cn (L.X.); 2530401032@stu.suda.edu.cn (Y.S.); 2 Suzhou Medical College, Soochow University, Suzhou 215123, China; 2330413002@stu.suda.edu.cn

**Keywords:** short-chain fatty acids, anti-aging, signaling pathways, aging-related diseases, intervention strategies

## Abstract

Aging is a systemic degenerative process that can lead to functional decline in multiple organs, such as skeletal muscles and the heart, and accelerates the overall aging process through organ-to-organ interactions mediated by metabolites such as short-chain fatty acids (SCFAs). SCFAs serve as a crucial link connecting intestinal health and anti-aging, and their levels and functions undergo significant changes with aging. However, current research lacks understanding of the downstream molecular mechanisms of SCFAs, and intervention methods are mostly limited to simple regulation. This article clarifies the intrinsic relationship between SCFAs and aging from a systemic perspective, analyzes their regulatory mechanisms through key signaling pathways, examines their roles in tissue barrier protection, the improvement of metabolic disorders, and immune regulation, and summarizes their therapeutic potential and diversified intervention strategies in aging-related diseases. The detailed molecular mechanisms by which SCFAs regulate aging are still unclear, and there are no precise intervention plans for different aging stages and organ damage. In the future, we need to utilize techniques such as single-cell sequencing and organ models to explore the regulation of aging cell fates, providing support for the development of metabolite-mediated personalized anti-aging intervention measures.

## 1. Introduction

**Figure 1:**
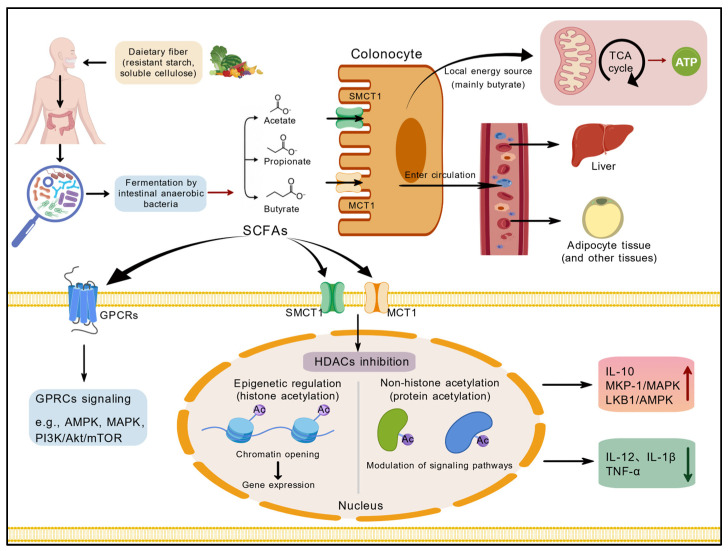
The production, transport, and core physiological functions of short-chain fatty acids (SCFAs). Abbreviations: AMPK, adenosine monophosphate-activated protein kinase; GPCRs, G protein-coupled receptors; HDACs, histone deacetylases; IL-10, interleukin-10; IL-12, interleukin-12; IL-1β, interleukin-1β; LKB1, liver kinase B1; MAPK, mitogen-activated protein kinase; MCT1, monocarboxylate transporter 1; MKP-1, mitogen-activated protein kinase phosphatase-1; PI3K/Akt/mTOR, phosphatidylinositol 3-kinase/protein kinase B/mammalian target of rapamycin; SMCT1, sodium-coupled monocarboxylate transporter 1; TCA cycle, tricarboxylic acid cycle; TNF-α, tumor necrosis factor-α. Different colors and shapes of bacteria are used for illustrative purposes only and do not represent specific bacterial taxa. Upward arrows indicate increased or upregulated effects, whereas downward arrows indicate decreased or downregulated effects. Created with BioGDP.com [5].

Short-chain fatty acids (SCFAs) are the main end products of dietary fiber fermentation by intestinal anaerobic bacteria, mainly including acetate, propionate, and butyrate [1]. After being produced in the colon, SCFAs are absorbed by colon epithelial cells through specific transporters such as sodium-coupled monocarboxylate transporter 1 and monocarboxylate transporter 1 [2]. A portion of SCFAs (mainly butyrate) is directly used as an energy source for colonocytes, participating in the tricarboxylic acid cycle to generate ATP; the remaining SCFAs enter the bloodstream and are transported to peripheral tissues, including the liver, adipose tissue, brain, and skeletal muscle, where they exert systemic regulatory effects [3]. In addition to energy supply, SCFAs can inhibit histone deacetylases (HDACs), thereby exerting dual regulatory effects: epigenetic regulation via histone acetylation, and direct modulation of protein function via non-histone acetylation. SCFAs can also activate intracellular signaling pathways by binding to G protein-coupled receptors (GPCRs) on the cell membrane [4] (Figure 1).

Aging is a progressive, systemic degenerative process characterized by multi-organ functional decline, chronic inflammation, metabolic disturbance, and impaired inter-organ communication [6,7,8]. Mounting evidence indicates that metabolites are core mediators of inter-organ crosstalk during aging [9], and the levels of gut microbiota-derived metabolites, especially SCFAs, decline significantly with physiological aging, which is closely associated with the onset and progression of multiple aging-related diseases [10].

At the cellular and molecular levels, existing studies have confirmed that SCFAs can reshape cellular function states by targeting multiple key aging-related signaling pathways. These pathways include adenosine monophosphate-activated protein kinase (AMPK), mitogen-activated protein kinase/extracellular signal-regulated kinase (MAPK/ERK), phosphoinositide 3-kinase/protein kinase B/mechanistic target of rapamycin (PI3K/Akt/mTOR), and nuclear factor kappa-light-chain-enhancer of activated B cells (NF-κB) pathways, all of which are core regulatory axes of the aging process [11,12,13,14,15].

At the systemic phenotypic level, existing studies have further verified the anti-aging effects of SCFAs: they can not only directly delay aging by maintaining the intestinal barrier, inhibiting chronic inflammation, and regulating metabolic homeostasis, but also indirectly reduce the risk of aging-related neurodegenerative diseases by modulating neural health through the gut–brain axis [16,17,18,19].

Although studies have confirmed the regulatory effects of SCFAs on individual aging characteristics and their corresponding signaling pathways, the holistic association between SCFAs and systemic aging remains fragmented. Most existing studies only focus on the local effects of SCFAs in a specific tissue or a single pathological process and have not yet systematically clarified the overall role of SCFAs in aging [10,20].

Therefore, this review aims to sort out the regulatory relationships among SCFAs, key aging-related signaling pathways, tissue functions, and aging phenotypes, clarify how SCFAs participate in aging progression at the molecular, cellular, tissue, and systemic levels, and further summarize the latest advances in SCFA-based anti-aging interventions, providing an integrated theoretical framework for future basic research and translational application in this field.

## 2. SCFAs Regulate the Aging Process

**Figure 2:**
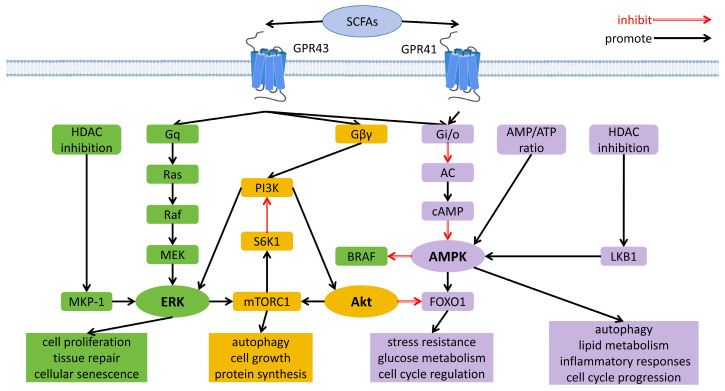
Molecular mechanisms of short-chain fatty acids (SCFAs) in anti-aging via multi-receptor signaling pathways. Abbreviations: AC, adenylate cyclase; Akt, protein kinase B; AMPK, adenosine monophosphate-activated protein kinase; BRAF, v-raf murine sarcoma viral oncogene homolog B1; cAMP, cyclic adenosine monophosphate; ERK, extracellular signal-regulated kinase; FOXO1, forkhead box protein O1; Gq, G protein q subunit; Gi/o, G protein i/o subunit; Gβγ, G protein βγ subunit; GPR41, G protein-coupled receptor 41; GPR43, G protein-coupled receptor 43; HDAC, histone deacetylase; LKB1, liver kinase B1; MEK, mitogen-activated protein kinase; MKP-1, mitogen-activated protein kinase phosphatase-1; mTORC1, mechanistic target of rapamycin complex 1; PI3K, phosphatidylinositol 3-kinase; Raf, rapidly accelerated fibrosarcoma; Ras, rat sarcoma viral oncogene homolog; S6K1, ribosomal protein S6 kinase 1.

As described in the previous section, SCFAs modulate four core aging-related signaling cascades (AMPK, MAPK/ERK, PI3K/Akt/mTOR, and NF-κB pathways) via two mechanisms: intracellular HDAC inhibition to regulate key pathway molecules (Figure 1) and GPCR-mediated transmembrane signal transduction via subtype-specific receptor binding and G protein coupling (Figure 2). The subtype-specific effects and quantitative parameters are summarized in Table 1.

The cross-regulatory network formed by these four pathways underlies the systemic anti-aging effects of SCFAs, including intestinal barrier protection, metabolic homeostasis improvement, and immune-inflammatory modulation (Section 2.2, Section 2.3 and Section 2.4), to reshape physiological homeostasis during aging.

### 2.1. The Molecular Mechanism of SCFAs in Regulating Aging

The four signaling pathways form an interconnected regulatory network with extensive crosstalk that collectively mediates the anti-aging effects of SCFAs (Figure 2). Specifically, the AMPK pathway functions as the central cellular energy sensor and regulates autophagy, lipid metabolism, inflammatory responses, and cell cycle progression. The MAPK/ERK pathway primarily controls cell proliferation, tissue repair, and cellular senescence. The PI3K/Akt/mTOR pathway coordinates cell growth, protein synthesis, autophagy, and forkhead box protein O1-mediated stress responses. Finally, the NF-κB pathway serves as the master regulator of age-related chronic inflammation and integrates signals from the other pathways.

#### 2.1.1. AMPK Pathway

AMPK is a central cellular energy sensor and a key anti-aging target of SCFAs [26,27,28,29,30]. SCFAs activate AMPK through multiple mechanisms.

First, acetate and propionate bind to GPR43, inhibit adenylate cyclase activity, reduce intracellular cAMP levels, and thereby relieve inhibition of AMPK [31] (Figure 2).

Second, butyrate activates the upstream kinase liver kinase B1, potentially through acetylation-dependent regulation [32]. Activated liver kinase B1 subsequently phosphorylates and activates AMPK, representing a major route linking SCFAs to cellular energy sensing.

Third, the metabolic activation pathway: SCFAs undergo β-oxidation during cellular metabolism to generate AMP, which increases the intracellular AMP/ATP ratio [33]. Elevated AMP promotes AMPK activation by facilitating its phosphorylation by upstream kinases [34].

Fourth, some studies suggest that butyrate may indirectly activate AMPK through adiponectin secretion, although the contribution of this mechanism remains unclear [35,36].

Activated AMPK regulates multiple aging-related processes, including autophagy, lipid metabolism, inflammatory responses, and cell cycle progression. However, the effects of SCFA-mediated AMPK activation are highly dependent on cell type and species. For example, AMPK activation inhibits adipogenic differentiation in 3rd subculture-L1 cells but does not prevent butyrate-induced expression of adipogenic transcription factors in porcine adipocytes [37,38,39]. In addition, acetate reduces lipolysis in human adipose-derived stem cell-derived adipocytes [28]. These observations highlight the need for caution when extrapolating findings from preclinical models to human aging.

#### 2.1.2. MAPK/ERK Pathway

The MAPK/ERK pathway is another major anti-aging signaling axis targeted by SCFAs and is primarily involved in cell proliferation, tissue repair, and cellular senescence [40].

SCFAs regulate MAPK/ERK signaling through two principal mechanisms.

First, SCFAs bind to GPR43 and activate Gq signaling, triggering the canonical Ras-Raf-MEK-ERK cascade (Figure 2). Through this pathway, SCFAs influence cell proliferation, differentiation, and tissue remodeling.

AMPK negatively regulates ERK signaling in part through direct inhibition of BRAF, linking energy sensing to mitogenic pathways [41].

Second, SCFAs may modulate MAPK/ERK signaling through HDAC inhibition. HDAC1–3 regulate the acetylation status of mitogen-activated protein kinase phosphatase-1, thereby affecting MAPK activity and inflammatory cytokine production [42].

The biological consequences of HDAC-mediated MAPK regulation are highly context dependent, and most evidence comes from rodent models, highlighting the need for further validation in human tissues [43,44,45,46].

#### 2.1.3. PI3K/Akt/mTOR Pathway

The PI3K/Akt/mTOR pathway is a major metabolic and longevity-associated signaling axis regulated by SCFAs and exhibits extensive crosstalk with both AMPK and MAPK/ERK pathways (Figure 2).

SCFAs activate PI3K/Akt signaling through GPR43-mediated pathways [47]. Activated Akt influences aging-related processes mainly through two downstream branches. First, forkhead box protein O1, a transcription factor involved in stress resistance, glucose metabolism, and cell cycle regulation, is inhibited by Akt but activated by AMPK, allowing SCFAs to balance cellular proliferation and stress adaptation [48]. Second, mTORC1, a key regulator of cell growth, protein synthesis, and autophagy, is activated by both Akt and ERK. Activated mTORC1 promotes protein synthesis through S6K1, while S6K1 provides negative feedback to restrain upstream signaling and maintain pathway homeostasis [49,50,51]. Collectively, SCFAs coordinate cellular growth, metabolism, and stress resistance through the forkhead box protein O1 and mTORC1 branches of the PI3K/Akt signaling pathway.

#### 2.1.4. NF-κB Pathway

NF-κB signaling is a central regulator of age-related chronic inflammation (inflammaging). During aging, persistent oxidative stress and mitochondrial dysfunction contribute to sustained NF-κB activation, which promotes nucleotide-binding oligomerization domain-like receptor family pyrin domain-containing 3 inflammasome activation and the production of pro-inflammatory mediators [52].

SCFAs suppress NF-κB-dependent transcription of inflammatory cytokines and chemokines [53]. In particular, butyrate enhances the expression of the anti-inflammatory cytokine IL-10 while reducing the production of IL-12, tumor necrosis factor-alpha, IL-1β, and nitric oxide [54,55,56] (Figure 1).

Multiple mechanisms contribute to NF-κB inhibition by SCFAs, including HDAC inhibition, GPR41/43-mediated signaling, and AMPK-dependent reduction of reactive oxygen species [57,58,59,60,61].

Notably, butyrate, propionate, and acetate inhibit NF-κB activity with a potency order of butyrate > propionate > acetate, which parallels their HDAC inhibitory capacities [53] (Table 1). This observation suggests that HDAC inhibition may represent a dominant mechanism underlying SCFA-mediated suppression of NF-κB signaling.

### 2.2. Protection of Organizational Barriers

Tissue barrier integrity is a critical defense against age-related systemic inflammation, with the intestinal barrier and blood–brain barrier being the primary targets of SCFAs.

The intestinal barrier prevents luminal antigens from entering the systemic circulation. Barrier dysfunction drives bacterial infiltration and immune dysregulation, a key initiating event in inflammatory bowel disease and age-related inflammation [62].

Butyrate is the dominant SCFA mediating intestinal barrier protection, acting via three core mechanisms: (1) it provides ~70% of cellular ATP for colonocytes via mitochondrial β-oxidation [62]; (2) it stabilizes hypoxia-inducible factor 1, a master regulator of barrier function [63]; (3) it upregulates tight junction protein expression via transcription factors signal transducer and activator of transcription 3 and specificity protein 1 [62]. These effects increase transepithelial electrical resistance across species and persist under inflammatory conditions [64,65,66,67]. Given the reduced abundance of butyrate-producing bacteria in inflammatory bowel disease patients, the intestinal barrier is a key therapeutic target of butyrate [68]. High concentrations of acetate also exert mild barrier-protective effects [62].

Blood–brain barrier disruption is closely linked to age-related neuroinflammation, neurodegeneration, and cognitive decline. As key gut–brain axis messengers, SCFAs are essential for maintaining blood–brain barrier integrity under pathological conditions.

**Figure 3:**
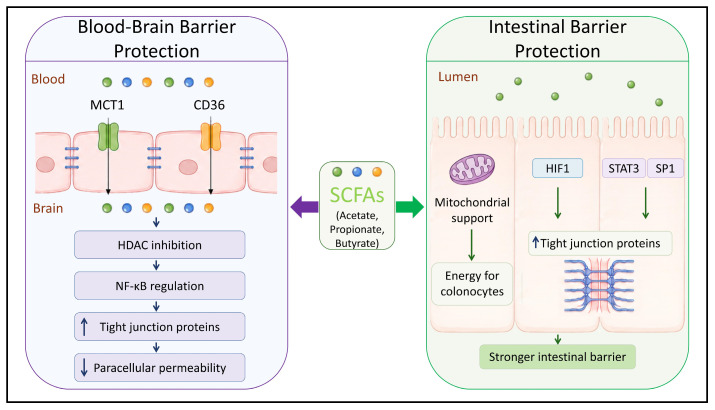
Roles of short-chain fatty acids (SCFAs) in barrier protection. Abbreviations: CD36, cluster of differentiation 36; HDAC, histone deacetylase; HIF-1, hypoxia-inducible factor 1; MCT1, monocarboxylate transporter 1; NF-κB, nuclear factor kappa B; SP1, specificity protein 1; STAT3, signal transducer and activator of transcription 3. Color coding is used to distinguish the major SCFAs: green represents butyrate, blue represents propionate, and orange represents acetate. Upward arrows indicate increased or upregulated effects, whereas downward arrows indicate decreased or downregulated effects.

SCFAs cross the blood–brain barrier mainly via monocarboxylate transporter 1 and fatty acid translocase cluster of differentiation 36. Their core protective effect is to reduce paracellular permeability by restoring tight junction proteins [52] (Figure 3). HDAC inhibition is the best-validated direct mechanism that regulates downstream NF-κB and nuclear factor erythroid 2-related factor 2 signaling to preserve barrier function. GPR41 has been detected in brain endothelial cells, but its functional role in blood–brain barrier protection remains unconfirmed.

### 2.3. Improvement of Metabolic Disorders

Metabolic disorders (such as imbalances in glucose and lipid metabolism) are important triggers of aging-related diseases [69]. During aging, dysregulated metabolic homeostasis mediates systemic pathological damage through multiple pathways, including promoting tumorigenesis, impairing neuronal function, and exacerbating chronic inflammation [41,69,70]. SCFAs regulate metabolic homeostasis through substrate supply and signal regulation pathways, thereby indirectly ameliorating aging-related pathological changes.

SCFAs mainly regulate glucose metabolism through propionate and butyrate. Butyrate activates intestinal gluconeogenesis genes via a cAMP-dependent mechanism, while propionate acts through the GPR41 receptor in the gut–brain axis [71]. Glucose produced enters the liver via the portal vein, signals the brain, suppresses hepatic glucose production, and enhances peripheral glucose uptake, improving glucose tolerance and insulin sensitivity. In the liver, SCFAs activate AMPK and inhibit gluconeogenic enzymes, including glucose-6-phosphatase and phosphoenolpyruvate carboxykinase [72]. Additionally, SCFAs can increase the expression of glucose transporter 4 in skeletal muscle cells and promote its translocation to the cell membrane, accelerating the absorption of glucose by myoblasts [73].

SCFAs maintain lipid homeostasis through multiple pathways. Propionate reduces hepatic cholesterol synthesis by inhibiting 3-hydroxy-3-methylglutaryl–coenzyme A reductase activity [74] and is negatively associated with serum low-density lipoprotein levels [75]. SCFAs also enhance hepatic cholesterol uptake, lowering plasma cholesterol [76]. In addition, SCFAs reduce food intake by stimulating leptin secretion and promoting the release of glucagon-like peptide-1 and peptide YY, thereby enhancing satiety through the gut–brain axis [77,78,79]. These effects collectively reduce appetite, body weight gain, and the risk of type 2 diabetes mellitus.

SCFAs regulate thermogenesis through uncoupling proteins 1-dependent and -independent pathways. Butyrate activates the AMPK/peroxisome proliferator-activated receptor gamma coactivator 1-alpha signaling axis to increase uncoupling proteins 1 expression, enhancing heat production [31,80]. It may also promote thermogenesis via calcium cycling, mitochondrial proton efflux, creatine cycling, and lipid turnover [80].

In obesity models, butyrate increases energy expenditure, improves insulin sensitivity, and reduces fat accumulation [81]. In humans, brown adipose tissue activity is lower in obese individuals. Activation of brown adipose tissue can increase diet-induced thermogenesis and reduce the respiratory quotient [82]. The direct effect of butyrate on human brown adipose tissue still needs further clinical verification.

SCFAs are important energy sources and precursors for lipid synthesis [83]. Acetic acid and butyric acid can be converted into acetyl coenzyme A, which participates in the tricarboxylic acid cycle for energy supply and can also be used to synthesize long-chain fatty acids or triglycerides for storage in adipose tissue. Although early studies suggested that excessive SCFAs might promote obesity [84], current evidence indicates that their beneficial effects predominate. Butyric acid production and the abundance of butyrate-producing bacteria (Bacteroidales) are reduced in patients with type 2 diabetes [85], and SCFA supplementation in high-fat diets can prevent diet-induced obesity and improve insulin sensitivity [86].

### 2.4. Immune and Inflammatory Regulation

SCFAs play a crucial role in restoring the immune–inflammatory balance and counteracting inflammaging during aging.

In the gut, SCFAs regulate intestinal immunity by activating GPR43 on group 3 innate lymphoid cells, promoting IL-22 secretion, maintaining epithelial barrier integrity, and enhancing host defense against bacterial infection. Loss of GPR43 impairs these protective effects and increases susceptibility to intestinal damage and infection [87].

SCFAs also exert anti-inflammatory effects by inhibiting NF-κB signaling and modulating immune cell function. Butyrate suppresses tumor necrosis factor-alpha and IL-6 production in adipose tissue and macrophages through histone deacetylase inhibition [88,89]. In addition, SCFAs reduce IL-8 production in neutrophils and promote neutrophil apoptosis via GPR43, thereby alleviating intestinal inflammation [89]. These effects help disrupt the cycle of inflammatory mediator release and tissue damage.

The immunomodulatory effects of SCFAs are cell type- and concentration-dependent. At low concentrations, SCFAs reduce neutrophil recruitment and inflammatory infiltration, whereas at higher concentrations, they promote regulatory T cell differentiation and IL-10 production, thereby enhancing immune tolerance [89,90].

However, the effects of SCFAs are not exclusively anti-inflammatory. Acetate has been associated with increased susceptibility to allergic asthma [91], and under certain conditions, SCFAs may activate inflammasome pathways through histone deacetylase inhibition and caspase-8-dependent signaling [92].

## 3. The Improvement Effect of SCFAs on Aging-Related Systemic Diseases

SCFAs, characterized by multi-target regulatory properties and cross-system actions, exert beneficial effects on aging-related diseases primarily through the gut–organ axis. Among these, growing evidence has highlighted their prominent roles in the nervous and cardiovascular systems, where they contribute to neuroprotection, vascular homeostasis, and the attenuation of age-related functional decline.

In the following sections, we systematically discuss the improvement effects of SCFAs in neurodegenerative and cardiovascular diseases, together with the underlying mechanistic insights, to provide a focused reference for anti-aging research and translational applications.

### 3.1. Neuroprotective Effects of Neurodegenerative Diseases: Focusing on the Gut–Brain Axis

SCFAs are key mediators of communication between the gut and the brain. They influence brain function via the vagus nerve (e.g., butyrate directly activating vagal afferent fibers), as well as immune and endocrine pathways, thereby regulating the hypothalamic–pituitary–adrenal axis and improving neurochemistry and behavior. Conversely, the brain modulates gut microbiota composition and SCFA production through intestinal motility, mucus secretion, and mucosal immune responses, forming a bidirectional regulatory loop. In addition, SCFAs help maintain gut–brain axis function by protecting the integrity of the blood–brain barrier [93].

In neurodegenerative diseases such as Alzheimer’s disease and Parkinson’s disease, SCFAs primarily exert effects by regulating neuroinflammation and neuronal function through the gut–brain axis, with evidence of gender-specific [94] and regional [95] differences. Reduced fecal SCFA levels are commonly observed in patients with Alzheimer’s disease, Parkinson’s disease, autism spectrum disorder, major depressive disorder, and multiple sclerosis [96], and this deficiency is associated with neurocognitive impairment in Alzheimer’s disease and Parkinson’s disease [95]. Long-term supplementation of SCFAs in APP/PS1 mice alleviates cognitive deficits by reducing amyloid-β deposition, inhibiting tau hyperphosphorylation, and restoring microbiota homeostasis [97]. In addition, rapamycin increases SCFA levels and enriches SCFA-producing gut microbiota, thereby reducing the risk of Alzheimer’s disease in apolipoprotein E4 carriers [98].

Brain aging is characterized by cognitive decline and structural changes, including hippocampal atrophy. SCFA supplementation improves cognitive function, reduces hippocampal atrophy and ventricular enlargement, and reshapes gut microbiota composition in aged mice [99]. In contrast, dietary fiber deficiency reduces SCFA production, leading to intestinal barrier dysfunction, decreased expression of calmodulin-dependent protein kinase II δ and synaptic proteins (including growth-associated protein 43 and synaptic vesicle protein 2C), as well as increased neuroinflammation and microglial-mediated synaptic phagocytosis, thereby exacerbating cognitive impairment [100]. Furthermore, SCFAs enhance astrocyte–neuron communication, including the glutamate–glutamine shuttle, primarily through their actions on astrocytes in vivo [97].

At the molecular level, SCFAs exert their effects through GPR41/43 signaling as well as via inhibition of HDACs, thereby increasing histone acetylation and modulating chromatin accessibility and transcriptional programs involved in immune and inflammatory responses [101]. However, excessive or dysregulated signaling may enhance microglial activation and amyloid pathology, suggesting that the effects of SCFAs are context-dependent.

### 3.2. Cardiovascular Diseases: Focusing on Vascular Homeostasis and Repair of Aging-Related Damage

**Figure 4:**
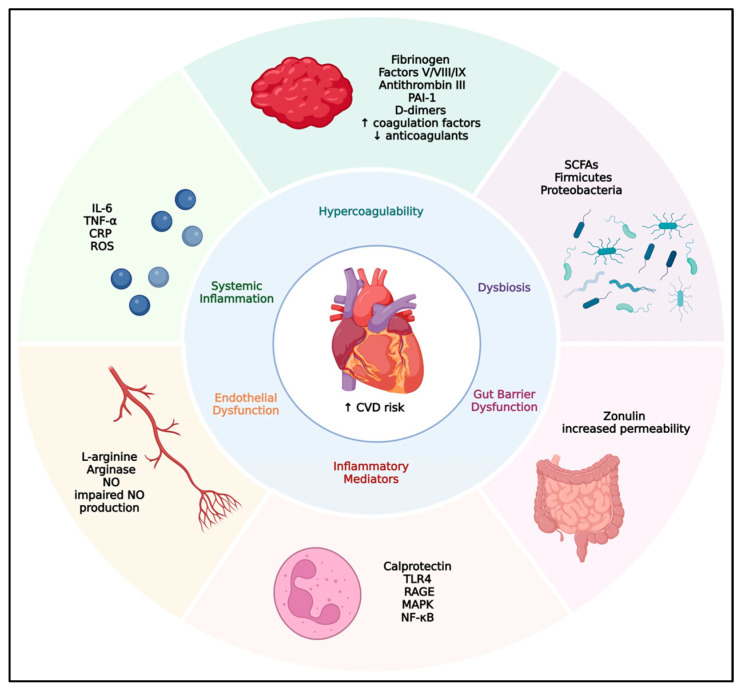
Roles of short-chain fatty acids (SCFAs) in cardiovascular diseases. SCFAs-producing bacteria depletion causes gut dysbiosis, which disrupts the intestinal barrier, triggers endotoxin translocation and systemic chronic inflammation, induces endothelial dysfunction and hypercoagulability, and ultimately increases cardiovascular disease risk. Abbreviations: CRP, C-reactive protein; CVD, cardiovascular disease; IL-6, interleukin-6; MAPK, mitogen-activated protein kinase; NF-κB, nuclear factor kappa B; NO, nitric oxide; NOS, nitric oxide synthase; PAI-1, plasminogen activator inhibitor-1; RAGE, receptor for advanced glycation end products; TLR4, Toll-like receptor 4. Reprinted from Ref. [103].

Cardiovascular diseases are major causes of morbidity and mortality in older adults. Emerging evidence suggests that SCFAs contribute to vascular homeostasis and may protect against age-related cardiovascular disorders [102] (Figure 4).

Hypertension is one of the most common age-related cardiovascular diseases. Most studies report reduced SCFA levels and a lower abundance of SCFA-producing bacteria in patients with hypertension [104,105], although some reports show elevated fecal SCFA levels, possibly due to impaired absorption and increased excretion [106]. SCFAs can lower blood pressure through multiple mechanisms. For instance, butyrate suppresses the renin–angiotensin system via the gut–brain axis [107], while propionate induces vasodilation through GPR41 [108].

Atherosclerosis is a chronic inflammatory disease. SCFAs exert protective effects by promoting regulatory T cell generation and improving endothelial function [109,110]. Butyrate reduces endothelial oxidative stress through the peroxisome proliferator-activated receptor delta/microRNA-181b pathway, while propionate lowers circulating cholesterol levels [110,111]. In aged mice, acetate supplementation restores arterial elasticity, improves endothelium-dependent vasodilation, and reduces systemic inflammation [112].

SCFAs are readily oxidized by failing hearts and may serve as an alternative energy source to ketone bodies [113]. In patients with chronic or congestive heart failure, the abundance of SCFA-producing bacteria and circulating levels of propionate and butyrate are reduced [114,115]. These findings highlight the potential of SCFAs in supporting cardiac metabolism, although the detailed mechanisms remain under investigation.

SCFAs may promote vascular repair after ischemic events. Acetate enhances lymphatic vessel formation and angiogenesis following ischemic stroke by increasing acetyl coenzyme A availability, correlating with vascular endothelial growth factor levels [116]. In myocardial ischemia–reperfusion injury, butyrate improves autonomic balance via gut–brain neural pathways [117], whereas propionate reduces injury by regulating angiotensin II levels through a GPR41-dependent pathway involving caveolin-1 and angiotensin-converting enzyme 2 [118].

SCFAs may also influence human immunodeficiency virus-related carotid atherosclerosis, though the underlying mechanisms are not fully understood [119]. Additionally, the gut–heart–mouth axis has received limited attention, offering new opportunities to explore SCFAs’ roles in cardiovascular health and age-related vascular dysfunction [120].

## 4. Anti-Aging Intervention Strategies Based on SCFAs

**Figure 5:**
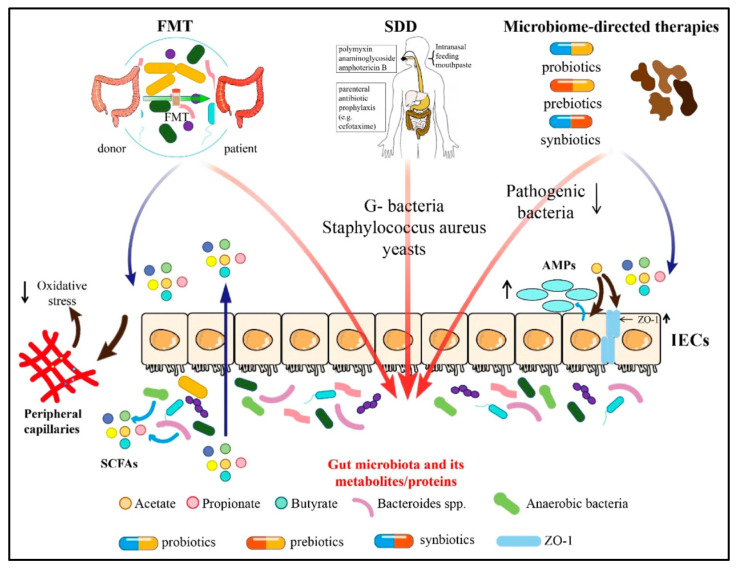
Short-chain fatty acid (SCFA)-based interventions for anti-aging. Fecal microbiota transplantation (FMT) improves intestinal oxidative stress and promotes SCFAs production to repair the intestinal barrier. Microbiome-directed therapies like probiotics, prebiotics, and synbiotics inhibit pathogenic bacteria, maintain intestinal epithelial cell (IEC) barrier function, and regulate intestinal microbiota imbalance. Abbreviations: SDD, selective digestive decontamination; AMPs, antimicrobial peptides; ZO-1, zonula occludens 1. Upward and downward arrows indicate increased and decreased levels or activities, respectively. Reprinted from Ref. [123].

SCFAs are signaling molecules mediating gut-organ axis communication. Their progressive decline with aging is closely associated with the development of multiple age-related diseases. SCFA-based intervention strategies, which modulate gut microbiota composition or directly supplement these metabolites, have emerged as a cutting-edge approach to delay aging and prevent age-related disorders. Multiple technical pathways have been developed, including dietary modulation, probiotic/prebiotic supplementation, postbiotic intervention, natural products, and fecal microbiota transplantation (FMT) (Figure 5). However, their efficacy exhibits significant individual heterogeneity, and clinical translation remains challenging [121,122].

### 4.1. Intervention Categories and Mechanistic Characteristics

To systematically compare the characteristics and translational potential of various intervention measures, we have compiled all reported SCFAs-based anti-aging intervention studies and constructed a detailed comparative framework (Table 2).

#### 4.1.1. Dietary Interventions

Diet represents the most fundamental and accessible strategy to modulate intestinal SCFAs levels, primarily by providing fermentable fiber substrates or optimizing dietary patterns to reshape microbial metabolic function [163]. The Mediterranean diet, high-fiber diet, and intermittent fasting have all been demonstrated to significantly increase SCFA levels in elderly populations and animal models, improve intestinal barrier function, reduce systemic inflammation, and delay cognitive decline [125,134]. Various natural foods, such as strawberries, purslane, soybeans, and rice bran are rich in fermentable fibers and phytochemicals that can specifically enrich SCFA-producing bacteria [129,131,132,164].

#### 4.1.2. Probiotic, Prebiotic, and Synbiotic Interventions

Probiotics increase SCFAs production by directly supplementing SCFA-producing strains or modulating microbiota structure [165].

The anti-aging effects of strains such as *Akkermansia muciniphila*, *Lactobacillus plantarum*, and *Lactobacillus rhamnosus* have been extensively validated [137,139,166].

Prebiotics such as inulin and galactooligosaccharides selectively promote the growth of SCFAs-producing bacteria, but their effects are highly dependent on the individual baseline microbiota composition [144,167].

Synbiotics, which combine the synergistic effects of probiotics and prebiotics, have shown superior efficacy to single agents in postmenopausal women’s bone protection and Alzheimer’s disease intervention [94,142].

#### 4.1.3. Postbiotic and Exogenous SCFA Supplementation

Postbiotics and exogenous SCFA supplementation can bypass gut microbiota differences to exert direct effects, offering the advantages of rapid onset and dose controllability. Mixed SCFAs and single-acetate, butyrate supplementation significantly improve cognitive function, reduce hippocampal atrophy, and reverse vascular aging in aged mice [100,112,168].

#### 4.1.4. Natural Products and Pharmacological Interventions

Multiple active ingredients from traditional Chinese medicine and natural extracts exert anti-aging effects through modulating the microbiota–SCFAs axis, including icariin, salvia miltiorrhiza polysaccharides, ginseng extract, and eucommia ulmoides leaves [150,151,153,154].

Classic anti-aging drugs such as rapamycin and vitamin E have also been shown to improve metabolism and cognitive function by enriching SCFA-producing bacteria [98,156,169].

#### 4.1.5. Fecal Microbiota Transplantation and Cell Therapy

FMT restores SCFAs production capacity by comprehensively reconstructing the intestinal microecosystem [170].

FMT from young healthy donors significantly improves cognitive function, reduces frailty, and promotes post-stroke recovery in aged mice [158].

Human umbilical cord mesenchymal stem cell therapy also induces beneficial changes in gut microbiota, increases the abundance of SCFA-producing bacteria, and alleviates aging-related DNA damage [160].

### 4.2. Precision Intervention Implementation Strategies

Addressing individual response heterogeneity is the primary barrier to clinical translation of SCFA-based interventions, as identical dietary inputs can produce up to 10-fold differences in SCFA production between individuals [171,172]. Microbial community-scale metabolic modeling (MCMM) has emerged as a transformative solution, enabling mechanistic, quantitative prediction of personalized SCFA production profiles directly from metagenomic data. This approach has been rigorously validated in vitro, ex vivo, and large human cohorts, providing causal explanations for intervention responses that statistical methods cannot match [173].

A standardized precision workflow has been established: baseline metagenomic sequencing to construct individual-specific MCMMs, simulation of intervention responses, stratified intervention assignment, and dynamic monitoring of clinical biomarkers. This framework effectively identifies and rescues intervention non-responders. In a cohort of 3129 individuals, 0.5% showed <20% butyrate increase with high-fiber diets, and 0.2% showed decreased production, but personalized combinatorial interventions identified by MCMM increased butyrate production by an average of 239–290%, significantly outperforming standard approaches [171,173,174,175].

Intervention precision is further refined by demographic and clinical stratification. Elderly individuals benefit more from butyrate-producing probiotics than prebiotics due to age-related bifidobacterial decline [129]. Women derive greater cognitive benefits from butyrate, while men show better metabolic responses to propionate [94,176,177,178]. Patients with chronic kidney disease should prioritize propionate; those with neurodegenerative disorders benefit from butyrate, and individuals with paradoxical inflammatory responses to high-fiber diets require targeted propionate-boosting interventions [124].

Importantly, traditional circulating SCFA measurements drastically underestimate true intestinal production. A 2023 stable isotope tracer study demonstrated that the gut-associated inaccessible pool accounts for 87–94% of total SCFA production, with generation rates 7–16 times higher and pool sizes 24–55 times larger than the accessible circulating pool [179]. Consistent with this, MCMM-predicted SCFA production rates show significantly stronger associations with 20 cardiometabolic and inflammatory biomarkers in the Arivale cohort than measured blood SCFA concentrations, reflecting the rapid colonic metabolism of these metabolites [180].

### 4.3. Limitations and Translational Challenges

Despite significant progress, SCFA-based anti-aging interventions face several unresolved challenges that must be addressed before widespread clinical implementation.

First, SCFAs exhibit context-dependent dual biological effects that complicate therapeutic application. While generally anti-inflammatory under homeostatic conditions, butyrate and propionate can activate the nucleotide-binding oligomerization domain-like receptor family pyrin domain-containing 3 inflammasome and promote pro-inflammatory cytokine release in the presence of TLR agonists or in established inflammatory environments [92,121,181,182]. This paradoxical effect explains why some individuals with pre-existing gut inflammation experience worsening symptoms with high-fiber diets, as unfermented fibers can directly activate inflammatory pathways, while impaired microbial metabolism fails to produce sufficient anti-inflammatory SCFAs. The threshold at which SCFAs transition from protective to harmful varies significantly between individuals and tissue types, further complicating dose optimization.

Second, the magnitude and direction of intervention responses are governed by a complex interplay of microbial, dietary, and host factors that remain incompletely understood. Even with advanced MCMM approaches, current models explain only 25–35% of the variance in butyrate and propionate production [173].

Key sources of unexplained variability include strain-level differences in metabolic capacity that are not captured by species-level metagenomic sequencing, inter-individual differences in host-derived substrates that serve as important carbon sources for gut microbes, pharmacokinetic variability in SCFA absorption, distribution, and metabolism, along with medication use and comorbidities that alter gut microbial function [119,173].

Third, long-term safety data for most SCFA-based interventions remain limited, particularly in elderly and diseased populations. While short-term studies have generally reported good tolerability, potential long-term risks include microbial dysbiosis from chronic prebiotic supplementation that may selectively enrich for specific taxa at the expense of overall microbial diversity; metabolic disturbances from excessive colonic fermentation that can lead to sustained elevations in circulating SCFAs associated with increased risk of insulin resistance and cardiovascular disease in some epidemiological studies; colorectal cancer risk as butyrate may promote tumor growth in established adenomas due to its role as a primary energy source for colonocytes; and drug interactions as SCFAs can modulate the expression and activity of hepatic drug-metabolizing enzymes, potentially altering the efficacy and toxicity of concurrent medications [119,124,183].

Fourth, current MCMM approaches have important technical limitations that restrict their clinical utility. Model accuracy depends heavily on the availability of high-quality, manually curated genome-scale metabolic models, which are lacking for many understudied gut taxa. Additionally, most existing models are based on microbiome data from Western populations, limiting their applicability to non-Western ethnic groups with distinct gut microbial compositions [173,184]. The computational complexity and expertise required to construct and run MCMMs also present barriers to widespread clinical adoption.

Fifth, preclinical studies in mice have inherent limitations that complicate translation to human aging. Many mouse studies are conducted in co-housed animals, making it difficult to isolate age-related effects from environmental and cage-specific microbial transmission. Standardized study designs, including individual housing, consistent fecal sample collection timing, and more frequent longitudinal sampling, will help eliminate residual variability and improve the reproducibility of findings. Furthermore, integrating multi-omics approaches such as metatranscriptomics and metabolomics will enable a more comprehensive understanding of the temporal dynamics of gut microbiome changes during aging [185].

Sixth, accurate quantification of SCFAs in complex biological matrices remains a technical challenge. Traditional methods often suffer from poor sensitivity, matrix interference, and an inability to simultaneously measure multiple SCFA species. However, recent advances in analytical techniques such as stable isotope labeling-assisted liquid chromatography–mass spectrometry (SIL-LC-MS) have shown promise for robust, simultaneous detection of SCFAs in various sample types, including biological specimens [186].

Finally, the development of effective delivery systems remains a major challenge for exogenous SCFA supplementation. Oral administration of free SCFAs results in rapid absorption in the upper gastrointestinal tract, with minimal amounts reaching the colon, where they exert their most beneficial effects. While colon-targeted formulations have shown promise in preclinical studies, their long-term safety and efficacy in humans remain to be established [187].

Addressing these challenges will require continued advances in microbial metabolic modeling, the development of more sophisticated delivery systems, and large-scale, long-term clinical trials that evaluate both efficacy and safety across diverse populations. Nevertheless, the ability to predict and modulate individual-specific SCFA production profiles represents a major step forward in the development of precision anti-aging interventions that harness the therapeutic potential of the gut microbiome.

## 5. Conclusions

SCFAs act as key metabolic factors that connect the gut microbiota and the regulation of systemic aging. By regulating gut–organ communication to counteract age-related physiological decline, they have become a pivotal target for anti-aging research and therapeutic development. SCFAs mainly exert aging regulatory effects by targeting key signaling pathways such as AMPK, MAPK/ERK, Akt/mTOR, and NF-κB. SCFAs exert anti-aging effects by reinforcing intestinal barrier function, attenuating systemic inflammation, and preserving metabolic homeostasis, while indirectly lowering the risk of neurodegenerative and other age-related disorders through inter-organ regulatory pathways, including the gut–brain axis. In terms of interventions, a diversified system has been established that focuses on modulating SCFAs production by gut microbiota, directly supplementing SCFAs metabolites, and fecal microbiota transplantation, offering feasible strategies to improve aging-related phenotypes. However, current research still faces major challenges. The regulatory mechanisms by which SCFAs modulate downstream molecules remain incompletely understood, limiting the development of precise targeted interventions. Moreover, most existing strategies focus on broadly elevating SCFAs levels, while stage- and organ-specific interventions for aging are yet to be established. To enhance the individualization of these intervention measures, it is necessary to conduct in-depth research on the molecular mechanism of SCFAs’ regulation of aging cell fate using technologies such as single-cell sequencing and organoid models in the future.

## Figures and Tables

**Table 1:** The Characteristics of Major Short-Chain Fatty Acid (SCFA) Targets and Receptors.

References	Target/ Receptor	Tissue/Cellular Expression Profile	Relative Potency Ranking of SCFA Subtypes	Quantitative Functional Parameters
[21,22,23]	HDAC	Ubiquitous expression throughout the body	Butyrate > Propionate >> Acetate	Butyrate: ~80% maximum inhibition efficiency on HDAC1/2; Propionate: ~60% maximum inhibition efficiency on HDAC; Acetate: no inhibitory activity at physiological concentrations
[24]	GPR43	Immune cells (neutrophils, macrophages), adipocytes, intestinal epithelial cells	Propionate ≥ Acetate ≥ Butyrate	EC50 of Acetate and Propionate: μM to low mM range
[25]	GPR41	Intestinal tract, adipose tissue, peripheral nervous system, pancreatic β-cells, immune tissues	Propionate ≥ Butyrate > Acetate	Higher affinity for propionate and butyrate, only weak activation of acetate at extremely high concentrations

Abbreviations: EC50, half maximal effective concentration; GPR41, G protein-coupled receptor 41; GPR43, G protein-coupled receptor 43; HDAC, histone deacetylase.

**Table 2:** Detailed Comparative Framework of Short-Chain Fatty Acid (SCFA)-Based Anti-Aging Interventions.

Intervention Category	Specific Intervention	Core Mechanism of Action	Primary Anti-Aging Efficacy	Level of Clinical Evidence	Target Population	Major Limitations	References
Dietary Interventions	Mediterranean diet	Increases the abundance of SCFA-producing bacteria, promoting acetate, propionate, and butyrate production	Improves the intestinal barrier, reduces systemic inflammation, lowers frailty risk, and delays cognitive decline	High (multiple large cohort studies + RCTs)	All healthy elderly and chronic disease high-risk populations	Requires long-term adherence, significant individual dietary compliance differences	[124,125]
	High soluble fiber diet (inulin, fructooligosaccharides)	Provides fermentation substrates for SCFAs-producing bacteria, enriches *Bifidobacterium* and *Faecalibacterium* genera	Increases intestinal SCFAs levels, reduces F/B ratio, and decreases the pro-inflammatory factor CXCL1	Moderate (extensive animal experiments + partial RCTs)	Constipation, metabolic syndrome, and prediabetes populations	High doses easily cause bloating and diarrhea, as well as reduced responsiveness in the elderly	[126,127,128]
	Strawberries (fresh/freeze-dried)	Enriches SCFAs-producing genera, including *Faecalibacterium* and *Prevotella*, reduces potential pathogens	Increases gut microbiota diversity, improves lipid metabolism, and reduces inflammation	Moderate (RCT)	Healthy elderly, mild cognitive impairment populations	Requires high intake and relatively high cost	[129]
	Purslane	Increases concentrations of acetate, propionate, butyrate, valerate, caproate, and total SCFAs, regulates the F/B ratio	Maintains intestinal morphological integrity and improves intestinal health	Low (animal experiments)	Elderly with reduced intestinal function	Lack of human clinical trial data	[130]
	Rice bran + tea seed oil	Increases SCFAs-producing *Clostridium* species and reduces endotoxin-producing Tannerellaceae	Alleviates gut–liver–brain axis imbalance, reduces neuroinflammation, and improves metabolic disorders	Low (animal experiments)	Postmenopausal women, metabolic syndrome populations	Lack of human clinical trial data	[131]
	Soybeans and daidzein	Enriches *Prevotella ruminicola*, increases SCFAs production, and promotes Treg cell secretion of IL-10	Extends healthy lifespan, reduces intestinal inflammation, and improves the intestinal barrier	Moderate (animal experiments + limited human studies)	Menopausal women, premature aging populations	Estrogen-like effects of soy isoflavones require attention	[132,133]
	Intermittent fasting (15 h/day)	Reshapes the gut microbiota, increases the abundance of SCFAs-producing bacteria	Induces cerebral ischemia tolerance, protects post-stroke brain function, effects independent of age and gender	Moderate (animal experiments + limited human studies)	Stroke high-risk populations, healthy elderly	Poor fasting compliance may cause hypoglycemia	[134]
	Methionine restriction (0.17%)	Increases the abundance of SCFA-producing bacteria, including Lachnospiraceae, *Roseburia*, and Ruminococcaceae	Reverses intestinal SCFA decline, reduces hippocampal oxidative stress and inflammation, and improves cognitive impairment	Low (animal experiments)	Cognitive decline in high-risk populations	Long-term restriction may cause malnutrition	[135]
Probiotic Interventions	*Lactobacillus amylolyticus* TD-3 + *Lactococcus lactis* MQ1-1	Activates the AMPK/MLCK pathway, increases tight junction protein expression, and enriches SCFA-producing bacteria	Alleviates aging-related intestinal barrier dysfunction, reduces oxidative stress, and inflammation	Low (animal experiments)	Elderly with impaired intestinal barrier	Lack of human clinical trial data	[136]
	*Akkermansia muciniphila*	Promotes SCFA production, improves metabolic health, reduces chronic inflammation, and maintains the blood–brain barrier	Improves insulin sensitivity, reduces neuroinflammation, and modulates Alzheimer’s disease progression	Moderate (extensive animal experiments + limited human studies)	Metabolic syndrome, Alzheimer’s disease high-risk populations	Live bacterial preparations require strict storage conditions	[137,138]
	*Lactobacillus plantarum* X7022	Improves fecal SCFAs content and increases the abundance of anti-inflammatory bacteria, including *Lactobacillus* and *Akkermansia*	Improves age-related memory impairment, alleviates cerebral oxidative stress and hippocampal inflammation, and protects neurons	Low (animal experiments)	Elderly with mild cognitive impairment	Lack of human clinical trial data	[139]
	*Lactobacillus plantarum* BFS1243	Partially reverses frailty-related microbiota dysbiosis and SCFAs reduction, upregulates irisin expression	Improves female frailty symptoms, reduces inflammatory markers, and enhances intestinal barrier integrity and physical endurance	Low (animal experiments)	Female pre-frail and frail populations	Lack of human clinical trial data	[140]
	*Lactobacillus plantarum* TWK10	Increases SCFA-producing bacteria abundance and total intestinal SCFA levels, and reverses pathogenic bacteria accumulation	Prevents age-related muscle strength loss, alleviates bone loss, and cognitive impairment	Low (animal experiments)	Sarcopenia, osteopenia in the elderly	Lack of human clinical trial data	[141]
Synbiotic Interventions	SBD111 (specific probiotics + prebiotics)	Regulates SCFAs levels and improves the inflammatory microenvironment	Reduces hip and femoral neck bone loss in postmenopausal obese/osteopenic women	High (multicenter RCT)	Postmenopausal obese or early osteopenic women	Bone density improvement was not statistically significant in the entire cohort	[142]
	*Akkermansia muciniphila* + galactooligosaccharides	Regulates cecal SCFAs concentrations and improves gut microbiota richness	Improves metabolic health in APP/PS1 mice, reduces neuroinflammation, and modulates Alzheimer’s disease symptoms	Low (animal experiments)	Alzheimer’s disease high-risk populations	Lack of human clinical trial data	[138]
	Synbio^®^ mixture	Increases fecal *Lactobacillus* and *Bifidobacterium* abundance, raises total SCFAs and butyrate levels	Reduces serum high-sensitivity C-reactive protein and modulates the gut microbiota	Moderate (RCT)	Healthy elderly	Small sample size, short follow-up duration	[143]
	*Lactobacillus plantarum* 69-2 + galactooligosaccharides	Increases cecal butyrate content by nearly 3-fold and activates the hepatic AMPK/SIRT1 pathway	Improves liver function, antioxidant capacity, and inflammatory status, alleviating aging	Low (animal experiments)	Premature aging populations	Lack of human clinical trial data	[26]
Prebiotic Interventions	Inulin	Selectively promotes the growth of SCFA-producing bacteria, including *Bifidobacterium*	Increases intestinal SCFA levels, improves the intestinal barrier, and prevents enteric nervous system atrophy	Moderate (extensive animal experiments + partial RCTs)	Constipation, spinal cord injury-related intestinal dysfunction populations	High individual variability; non-responders exist	[122,126]
	Galactooligosaccharides	Synergistically enhances SCFAs production with probiotics	Improves gut microbiota, protects against skin photoaging	Moderate (animal experiments + limited human studies)	Skin aging populations	High doses may cause gastrointestinal discomfort	[144,145]
	HAMSAB (high-amylose maize starch + sodium butyrate)	Enhances serum acetate and butyrate production	Improves glycemic control in type 1 diabetes patients and maintains β-cell function	Moderate (animal experiments + limited human studies)	Type 1 diabetes patients	Insufficient long-term safety data	[146]
	Particle-size-reduced wheat bran	Increases fasting serum acetate and total SCFAs concentrations in obese subjects	No clear health benefits observed	Moderate (RCT)	Obese populations	No improvement in metabolic parameters was observed	[147]
Postbiotic/SCFAs Supplementation	Mixed SCFAs (acetate:propionate:butyrate = 60:20:20)	Directly supplements SCFAs, modulates the gut microbiota, and activates relevant signaling pathways	Improves cognitive deficits in aged mice, reduces hippocampal atrophy, and ameliorates inflammaging	Moderate (extensive animal experiments)	Cognitive impairment, inflammaging populations	Poor taste; significant gastrointestinal irritation	[99,148]
	Acetate	Improves vascular endothelial function and inhibits the early growth response-1 signaling pathway	Reverses increased aortic stiffness in aged mice and restores carotid artery endothelium-dependent relaxation	Moderate (animal experiments + limited human studies)	Atherosclerosis high-risk populations	Low bioavailability, strong first-pass metabolism	[112,149]
	Butyrate	Inhibits HDAC, activates the AMPK pathway, and promotes neuronal plasticity	Improves cognition, inhibits inflammation, and protects neurons, promoting myocyte proliferation	Moderate (extensive animal experiments + limited human studies)	Cognitive impairment, sarcopenia populations	Strong colonic first-pass metabolism, low systemic exposure	[45,92,99]
	Pasteurized *Akkermansia muciniphila*	Promotes SCFA production and activates the TLR2 signaling pathway	Improves metabolic health, reduces fat accumulation, and increases GLP-1 secretion	Moderate (animal experiments + limited human studies)	Metabolic syndrome populations	Mechanism of action not fully elucidated	[137]
Natural Product Interventions	Icariin	Reshapes the microbial composition, enriches SCFA-producing genera, and upregulates SCFA content	Alleviates d-galactose-induced cognitive impairment, improves mitochondrial function	Low (animal experiments)	Elderly with mild cognitive impairment	Lack of human clinical trial data	[150]
	Salvia miltiorrhiza polysaccharides	Increases intestinal SCFA content and regulates gut microbiota structure	Improves working memory impairment in aged mice, reduces oxidative stress and inflammation	Low (animal experiments)	Cognitive decline populations	Lack of human clinical trial data	[151]
	White mushroom polysaccharides	Increases α-diversity, SCFA levels, and *Bacteroides* abundance	Improves motor ability, spatial memory, and recognition memory, and reduces brain inflammation	Low (animal experiments)	Cognitive decline in populations	Lack of human clinical trial data	[152]
	Eucommia ulmoides leaf aqueous extract	Increases fecal SCFAs content, protects intestinal barrier integrity	Alleviates colitis-associated cognitive dysfunction, inhibits the JNK/TLR4 signaling pathway	Low (animal experiments)	Inflammation-related cognitive impairment populations	Lack of human clinical trial data	[153]
	Ginseng extract	Increases colonic acetate, propionate, and butyrate content, and activates the Wnt/β-catenin pathway	Improves intestinal function, regulates intestinal stem cell function, and protects intestinal health	Low (animal experiments)	Elderly with reduced intestinal function	Lack of human clinical trial data	[154]
	Rhubarb extract	Promotes butyrate-producing bacteria and short-chain fatty acid production	Alleviates chronic constipation in middle-aged adults	Moderate (RCT)	Middle-aged and elderly with chronic constipation	Long-term use may cause intestinal dependence	[155]
	Vitamin E	Increases the relative abundance of SCFA-producing bacteria and bile acid-metabolizing bacteria	Reduces low-density lipoprotein cholesterol levels, and improves lipid metabolism	Moderate (RCT)	Elderly with dyslipidemia	High doses may increase bleeding risk	[156]
Pharmacological Interventions	Rapamycin (1 mg/day)	Increases SCFA levels and enriches the gut microbiota associated with SCFA production	Improves cerebral blood flow in APOE4 carriers, reduces inflammation, and enhances lipid metabolism	Moderate (clinical trial)	Cognitively normal middle-aged APOE4 carriers	Immunosuppression risk; unclear long-term safety	[98]
	Berberine	Regulates gut microbiota balance, affects SCFA-related metabolism	Lowers blood glucose, improves insulin resistance	High (extensive clinical application)	Type 2 diabetes patients	Significant gastrointestinal adverse reactions	[157]
Fecal Microbiota Transplantation	FMT from young, trained donors	Reshapes the aged microbiota, increases butyrate and valerate levels, and reduces intestinal permeability	Improves cognitive function and synaptic plasticity, and reduces neuroinflammation	Low (animal experiments)	Elderly with cognitive impairment	Strict donor screening is required; infection transmission risk	[158]
	FMT from young healthy donors	Restores intestinal microecology, increases SCFA levels, and inhibits the TLR4/NF-κB pathway	Reduces frailty symptoms in aged mice, improves muscle mass, and intestinal barrier	Low (animal experiments)	Frail elderly	High individual variability in colonization success rate	[159]
	FMT from daidzein-treated donors	Transfers SCFA-producing microbiota characteristics	Rejuvenates aging intestine, extends lifespan in progeroid mice	Low (animal experiments)	Premature aging populations	Lack of human clinical trial data	[132]
Cell Therapy	Human umbilical cord mesenchymal stem cells	Induces beneficial changes in gut microbiota, increases the abundance of SCFA-producing bacteria	Alleviates aging-related DNA damage, improves motor coordination, reduces anxiety	Low (animal experiments)	Progeria syndrome populations	Technically complex, high cost, ethical controversies	[160]
Exercise Interventions	Combined aerobic exercise	Increases the abundance of *Bifidobacterium* and *Oscillibacter* genera and raises fecal butyrate levels	Improves gut microbiota and alleviates age-related microbiota dysbiosis	Moderate (RCT)	Sedentary elderly	Requires long-term adherence and poor compliance	[161]
	Resistance training (10 weeks)	No significant effects on gut microbiota, SCFAs, or gastrointestinal integrity markers	Increases muscle mass and strength	Moderate (RCT)	Sarcopenia populations	Limited SCFA-modulating effects	[162]

Abbreviations: AMPK, AMP-activated protein kinase; APOE4, apolipoprotein E ε4 allele; APP/PS1, amyloid precursor protein/presenilin-1; CXCL1, C-X-C motif chemokine ligand 1; F/B ratio, Firmicutes/Bacteroidetes ratio; FMT, fecal microbiota transplantation; GLP-1, glucagon-like peptide-1; HDAC, histone deacetylase; HAMSAB, high-amylose maize starch and sodium butyrate; IL-10, interleukin-10; JNK, c-Jun N-terminal kinase; MLCK, myosin light chain kinase; NF-κB, nuclear factor kappa B; RCT, randomized controlled trial; SIRT1, sirtuin 1; TLR2, Toll-like receptor 2; TLR4, Toll-like receptor 4; Treg, regulatory T cell.

## Data Availability

No new data were created or analyzed in this study.

## Abbreviations

The following abbreviations are used in this manuscript:

Akt/mTOR	protein kinase B/mechanistic target of rapamycin
AMPK	adenosine monophosphate-activated protein kinase
FMT	fecal microbiota transplantation
GPR43	G-protein coupled receptor 43
GPR41	G-protein coupled receptor 41
GPCRs	G-protein coupled receptors
HDAC	histone deacetylase
MAPK/ERK	mitogen-activated protein kinase/extracellular signal-regulated kinase
MCMM	microbial community-scale metabolic modelling
NF-κB	nuclear factor kappa-light-chain-enhancer of activated B cells
PI3K	phosphoinositide 3-kinase
SCFAs	short-chain fatty acids
